# Benefits of bilateral cochlear implantation in children: a systematic review and meta-analysis

**DOI:** 10.1007/s00405-026-10302-z

**Published:** 2026-05-21

**Authors:** Anastasia Zhuchkova, Joan Remacha, Marta Sandoval, Alberto Codina, Francesc Larrosa, Manuel Bernal-Sprekelsen

**Affiliations:** 1 Dept. of Surgical Specialties, University of Barcelona Medical School, Casanova 143, Barcelona, 08036 Spain; 2 Faculty of Medicine, Departament d´Especialitats Quirùrgiques, Hospital Clinic, University of Barcelona, c/Casanova 143, Barcelona, 08036 Spain

**Keywords:** Bilateral cochlear implantation, Pediatric cochlear implants, Speech perception in noise, Sound localisation, Language development, Hearing outcomes, Systematic review, Meta-analysis

## Abstract

**Objective:**

To systematically evaluate the benefits and risks of bilateral cochlear implantation (BiCI) in children compared with unilateral cochlear implantation (UniCI), with a focus on auditory outcomes, language development, and potential complications.

**Methods:**

A systematic review and meta-analysis was conducted following PRISMA guidelines and registered in PROSPERO (1128982). Searches were performed in PubMed, ScienceDirect, Scopus, and the Cochrane Library for studies published between 2015 and June 2025, with selective inclusion of earlier high-quality pediatric studies. Eligible studies included children with congenital or early-onset bilateral deafness who received BiCI or UniCI. Primary outcomes were speech perception (especially in noise), sound localisation, and spoken language development. Secondary outcomes included quality of life and adverse effects. Random-effects meta-analyses were performed using standardised mean differences (SMD).

**Results:**

Fifty studies were included in the qualitative synthesis and 20 in the meta-analyses. BiCI provided significantly better outcomes in speech perception in noise (SMD = + 0.70, *p* < 0.001) and sound localisation (SMD = + 0.74, *p* < 0.001), with consistent benefits across study designs and populations. Language development also favoured BiCI (SMD = + 0.65 for receptive vocabulary). Earlier implantation and shorter inter-implant intervals were associated with larger benefits. Surgical and vestibular complications were infrequent and did not exceed those reported for UniCI. Quality-of-life ratings were consistently higher in BiCI users.

**Conclusion:**

Bilateral cochlear implantation offers a significant but variable advantage over unilateral implantation in children, particularly in noisy environments, spatial hearing, and language acquisition. While language outcomes generally favour BiCI, results show greater heterogeneity compared to auditory measures. Benefits are most robust with early and simultaneous implantation. Although BiCI is a safe and effective intervention, the observed methodological and clinical heterogeneity underscores the need for standardized longitudinal assessments.

**Supplementary Information:**

The online version contains supplementary material available at https://doi.org/10.1007/s00405-026-10302-z.

## Introduction

Children born with profound bilateral hearing loss face significant challenges in speech and language development if left untreated. Congenital deafness affects approximately 1 to 3 per 1,000 live births worldwide, and among permanent cases identified through newborn screening programmes, bilateral hearing loss constitutes the larger proportion of clinically significant impairment [1]. Cochlear implants (CIs) have substantially improved outcomes; however, unilateral implantation (UniCI) does not restore binaural hearing, limiting performance in real-world noisy environments, reducing the ability to perceive soft speech, localize sounds accurately, and acquire language incidentally [2, 3].

To address these limitations, bilateral cochlear implantation (BiCI) is increasingly becoming the standard of care in pediatric deafness. BiCI leverages binaural cues—such as head shadow, summation, and binaural squelch—to enhance speech perception in noise and spatial hearing [4, 5]. It also provides more complete auditory input during critical periods of neurodevelopment [2, 6].

These advantages must be weighed against potential surgical and vestibular risks. A second CI requires an additional surgery (or a longer simultaneous bilateral procedure), which may increase perioperative complications. There are also concerns about vestibular effects, as implantation may disrupt inner ear receptors [7]. Several narrative and systematic reviews have previously examined pediatric bilateral cochlear implantation, including Sarant et al. [8], Litovsky & Gordon [3] and Thomas et al. [9]. These reviews concluded that BiCI improves spatial hearing and speech-in-noise, but were limited by small cohorts, heterogeneous outcome measures, and a lack of contemporary implant technology. Since 2018, more than 25 pediatric cohort studies and multiple multicentre datasets have been published, many using modern processors and earlier implantation ages. No meta-analysis incorporating these newer datasets currently exists. Given recent studies involving larger cohorts and improved technology, a systematic review is now warranted.

This systematic review and meta-analysis evaluates the advantages and disadvantages of bilateral cochlear implantation (BiCI) in children compared to unilateral implantation (UniCI). The primary outcomes assessed include speech perception, sound localisation, and language development. Secondary outcomes comprise surgical and vestibular risks, as well as quality of life. The aim is to quantify the benefits, clarify potential risks, and inform clinical decision-making regarding pediatric BiCI. Where possible, simultaneous and sequential bilateral implantation and inter-implant delay were analysed separately as key modifiers of outcome.

## Materials and methods

This study was registered at PROSPERO (CDR 1128982).

The clinical question of this systematic review was structured according to the PICO framework. The population (P) comprised children with congenital or early-onset bilateral severe-to-profound sensorineural hearing loss. The intervention (I) was bilateral cochlear implantation, either simultaneous or sequential. The comparator (C) was unilateral cochlear implantation. The outcomes (O) included speech perception in noise, sound localisation, spoken language development, quality of life and functional hearing outcomes, as well as device- and surgery-related complications.

This review followed PRISMA guidelines and focused on pediatric patients with bilateral congenital or early-onset deafness who received cochlear implants (CI). Early-onset deafness was defined as onset before 3 years of age, consistent with auditory critical-period literature. The intervention was bilateral cochlear implantation (BiCI), either simultaneous or sequential, compared to unilateral implantation (UniCI). Both between-group studies (BiCI vs. UniCI) and within-subject designs (same children tested with one vs. two implants) were included. Inter-implant interval was extracted for all sequential BiCI cases and categorised as short (< 12 months), intermediate (12–36 months), or long (> 36 months) for subgroup analyses. Studies lacking original data, without a comparator group, or limited to adult populations were excluded.

Primary outcomes included speech perception (especially in noise), sound localisation accuracy, and spoken language development (standardised test scores or receptive vocabulary measures). Secondary outcomes comprised quality of life and adverse effects, including surgical and vestibular complications. Major complications were defined as requiring revision surgery, explantation, or hospitalisation; minor included transient vertigo, wound issues, or otitis.

The PubMed search strategy was: (“cochlear implant” AND (bilateral OR unilateral) AND child OR pediatric) AND (speech OR localization OR language OR vestibular OR quality of life). Equivalent syntax was adapted for Scopus, ScienceDirect and Cochrane. The main search spanned publications from 2015 to June 2025. Bibliographies of included articles were manually screened. No language restrictions were applied.

Screening was conducted using the Rayyan.ai platform. From each study, data were extracted on study design, participant characteristics, intervention details, and outcome measures. Key variables included sample size, age at first and second implantation, inter-implant interval (for sequential BiCI), and CI duration at testing. For between-group studies, means, standard deviations (SDs), and group sizes were recorded. For within-subject designs, data were extracted for both one- and two-implant conditions. Where studies reported outcomes at multiple time points, the latest follow-up was prioritised, unless earlier time points allowed better comparability across studies.

Most included studies were observational. An adapted version of the Newcastle–Ottawa Scale was used to assess the risk of bias, focusing on cohort matching (by age and deafness duration), blinding of assessors (although uncommon, objective measures reduced bias), and completeness of follow-up. Studies scoring ≥ 7 on the Newcastle–Ottawa Scale were considered high quality. Selected high-quality studies from 2010 to 2015 were also included in cases where recent data were limited. Within-subject studies were also reviewed for order effects and learning bias; most randomised the test order to mitigate this. No randomised controlled trials (RCTs) were identified, likely due to ethical and logistical constraints in pediatric CI research.

Meta-analyses were conducted for outcome domains reported by at least three studies using comparable metrics. Standardised mean differences (SMDs) were used for continuous outcomes, including speech-in-noise perception, localisation error, and receptive vocabulary scores (e.g., Peabody Picture Vocabulary Test, PPVT). Random-effects models (DerSimonian–Laird method) were employed to account for inter-study variability. Heterogeneity was assessed using the I² statistic and interpreted as low (< 25%), moderate (25–50%), or high (> 50%). Funnel plots were generated to assess publication bias when ten or more studies contributed to an outcome (applied to speech-in-noise data).

All statistical analyses were performed using Meta-Essentials version 7 [10]. Statistical significance was set at *p* < 0.05 (two-tailed), and results are reported with 95% confidence intervals.

## Results

**Fig. 1:**
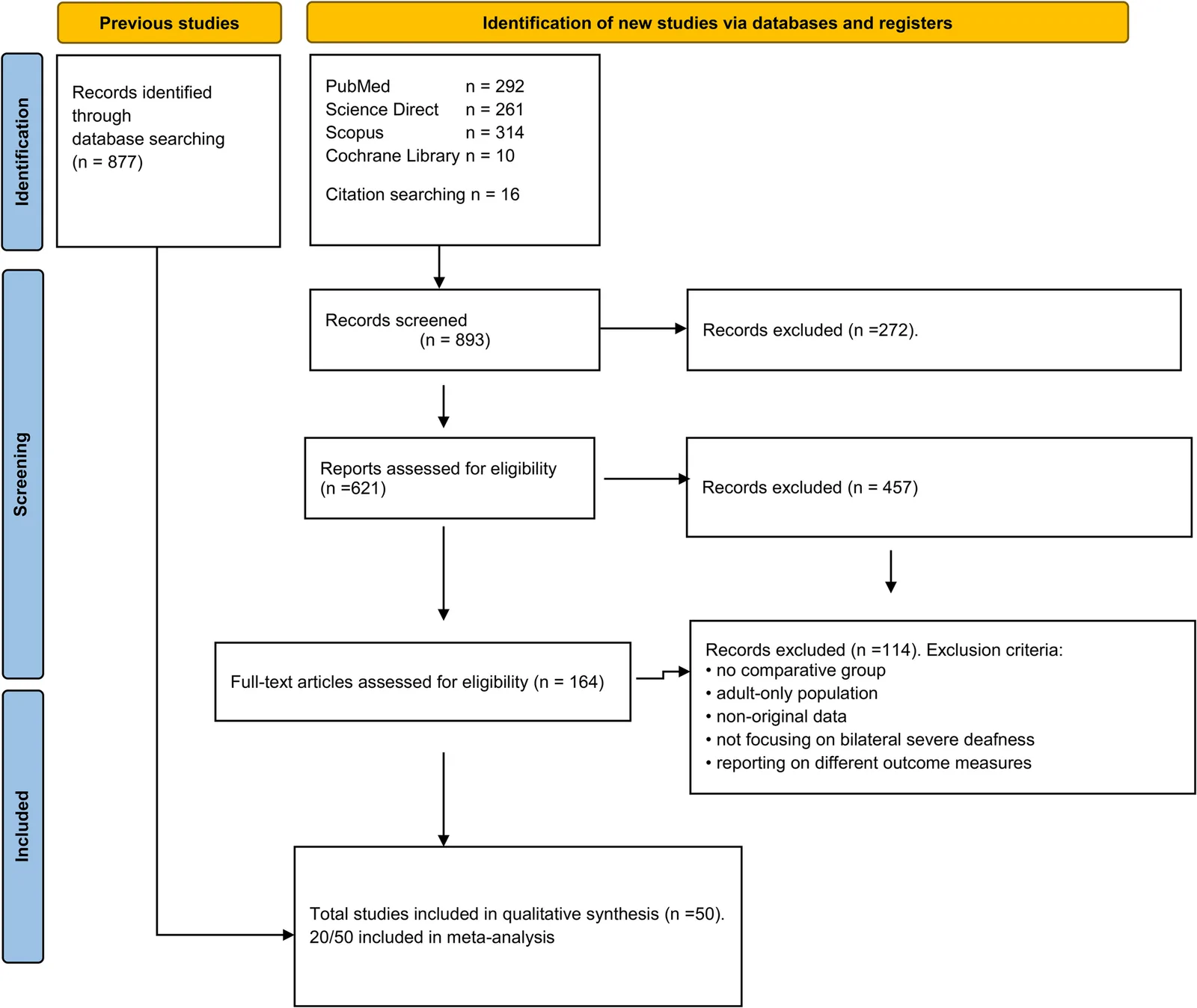
Illustrates the study selection process in a PRISMA flow diagram

After deduplication, 621 articles remained. Of these, 457 were excluded based on title and abstract screening, and 164 underwent full-text review. Fifty studies met inclusion criteria for the qualitative synthesis, and 20 were eligible for meta-analysis. The study selection process is shown in Fig. 1. Meta-analysed studies are summarized in Table 1.

**Table 1:** Complete list and summary of studies included in the meta-analyses. Separated by blue lines by groups according to outcomes assessed

Study	Year	Study Design	Sample Size (*N*)	Age (mean or range)	Outcomes Assessed	Measurement Tools	Effect Size (SMD, 95% CI)
Asp et al. [11]	2022	Prospective longitudinal cohort	18 (BiCI only)	Implant age: 0.42–2.3 years; test age: 0.58–3.6 years	Sound localization	12-loudspeaker horizontal localization task; Error Index (EI)	0.58 (0.19, 0.96)
Galvin & Mok [41]	2016	Within-subject cohort	14 (within-subject)	Children (range not stated)	Speech perception in noise	Custom sentence-in-noise test	0.77 (0.17, 1.37)
Cuda et al. [18]	2014	Cross-sectional cohort	30 UniCI	8–17 months	Speech development	MacArthur-Bates Communicative Development Inventory (MCDI) at 36 months.	0.88 (0.65, 1.10)
Ikiz Bozsoy & Yücel [5]	2024	Cross-sectional cohort	50 (BiCI = 25, UniCI = 25)	6–9 years	Speech perception in noise	HINT-C (Turkish)	1.10 (0.51, 1.69)
Jacobs et al. [12]	2016	Cross-sectional cohort	49 (BiCI = 18, UniCI = 31)	Mean ~ 8–12 years	Speech perception in noise	AAST (Adaptive Auditory Speech Test)	0.93 (0.32, 1.54)
Johnstone et al. [13]	2010	Within-subject cohort	16 (within-subject)	Mean ~ 8 years	Speech perception in noise	Custom spatialized speech test	0.74 (0.19, 1.29)
Li et al. [22]	2023	Within-subject cohort	20 (within-subject)	5–12 years	Speech perception in noise	Mandarin HINT or equivalent	0.66 (0.18, 1.14)
Puechmaille et al. [24]	2020	Within-subject cohort	63 (within-subject)	Mean ~ 6–10 years	Speech perception in noise	Custom protocol, 3 conditions (C1), C1 + HA, BiCI)	0.25 (0.00, 0.50)
Sparreboom et al. [6]	2011	Cross-sectional cohort	38 (BiCI = 29, UniCI = 9)	4–6 years	Speech perception in noise	Sentence in noise (% correct)	1.20 (0.40, 2.00)
Torkildsen et al. [25]	2019	Cross-sectional cohort	26 (BiCI = 12, UniCI = 14)	Mean 8.5 years	Speech perception in noise	HINT (Norwegian)	0.63 (−0.16, 1.42)
Grieco-Calub & Litovsky [21]	2010	Cross-sectional	38 (19 BiCI, 19 UniCI)	6.9 years (mean)	Sound localization (RMS error)	Speaker-array based RMS error test	0.94 (0.63, 1.26)
Litovsky et al. [48]	2016	Within-subject cohort	13 (within-subject)	5–9 years (range)	Sound localization (RMS error)	Speaker-array RMS (BiCI vs. UniCI condition)	0.49 (0.12, 0.86)
Misurelli et al. [15]	2016	Within-subject cohort	13 (within-subject)	5–11 years (range)	Sound localization (RMS error)	Custom 7-speaker array RMS task	0.66 (0.32, 0.99)
Polonenko et al. [50]	2018	Within-subject cohort	9 (within-subject)	4.7 years (mean)	Sound localization (RMS error)	Multi-speaker localization test	0.40 (−0.04, 0.83)
Vincent et al. [16]	2012	Cross-sectional	18 (9 BiCI, 9 UniCI)	7–11 years (range)	Lateralization (left/right discrimination)	% correct discrimination	1.97 (1.30, 2.63)
Jang et al. [17]	2019	Cross-sectional	45 (22 BiCI, 23 UniCI)	4–7 years (range)	Sound localization (angular deviation)	Angular deviation of head orientation	0.35 (0.02, 0.67)
Sarant et al. [2]	2014	Cohort (between-subject)	91 (UniCI = 24, BiCI = 67)	8 years (PPVT assessed at age 8)	Receptive vocabulary	PPVT-4	0.73 (0.25, 1.21)
Wie et al. [26]	2020	Cohort (between-subject)	30 (UniCI = 15, BiCI = 15)	Not specified (school-age)	Receptive vocabulary	PPVT-4	0.67 (−0.07, 1.41)
Colletti et al. [29]	2011	Cohort (between-subject)	40	10-year follow-up	Receptive vocabulary	Italian PPVT-R	0.60 (−0.03, 1.23)
Guerzoni et al. [31]	2020	Cohort (between-subject)	89 (UniCI = 45, BiCI = 27)	Mean 9.86 years (SD 1.98)	Receptive vocabulary	Italian PPVT	0.60 (0.11, 1.09)

*SMD *standardized mean deviation;* CI *= confidnce interval; *UniCI* =unilatyeral cochlea implant; *BiCI *= bilateral cochlea implant; *RMS* = root mean square error;* PPVT*‑*4*: Peabody Picture Vocabulary Test, Fourth Edition; *PPVT‑R* = Peabody Picture Vocabulary Test – Revised.

A comprehensive overview of all 50 studies included in the qualitative synthesis, including study design and key findings, is provided in Supplementary Table 1.

### Study sample characteristics

Sample sizes varied widely from 10 to 30 children in single-centre studies to large population cohorts. Age at implantation ranged from 5 to 6 months (first implant) to 8–10 years for second implants in sequential cases. Approximately half of bilateral cochlear implantation (BiCI) cases were simultaneous, with the remainder implanted sequentially. Reported inter-implant delays ranged from a few months to over five years and were described heterogeneously across studies using means, medians, ranges, or delay categories. Across studies reporting delay categories, most children received their second implant within 1–3 years, although several cohorts included long-gap sequential cases exceeding 5 years. Due to lack of patient-level data, pooled proportions of children in each delay category could not be calculated.

### Speech perception outcomes

**Fig. 2:**
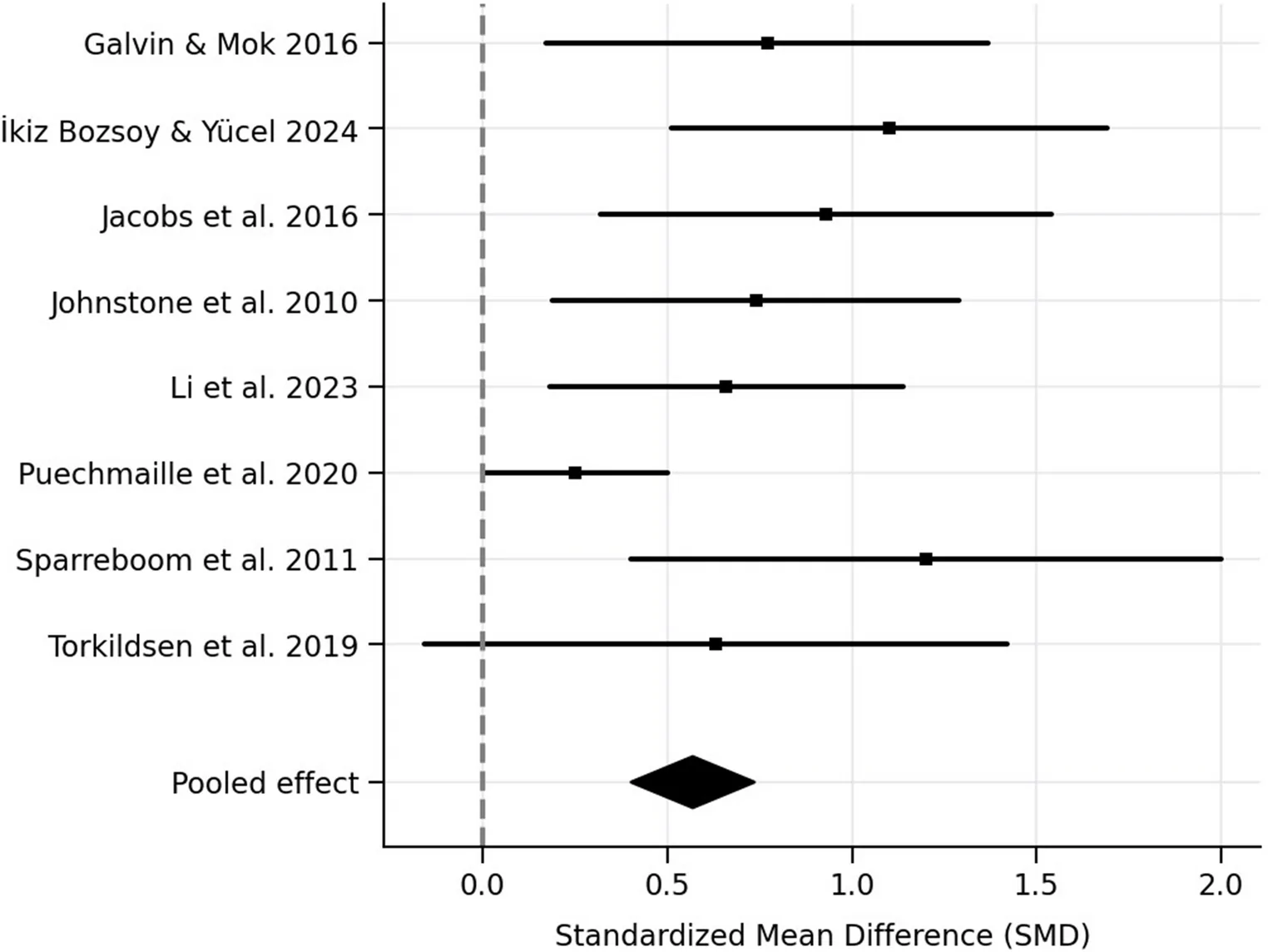
Forest plot of standardized mean differences (SMDs) comparing bilateral cochlear implantation (BiCI) with unilateral cochlear implantation (UniCI) for **speech perception in noise**. Only studies directly assessing speech recognition under noisy listening conditions were included. Positive SMD values indicate superior performance with bilateral implantation. The pooled effect (diamond) represents the fixed‑effect meta‑analytic estimate. Pooled estimate: SMD = 0.57 (95 % CI = 0.40 to 0.74)

Ten studies [11–20] contributed data to the meta-analysis (Fig. 2). Large cohort studies consistently demonstrated improved speech-in-noise performance following second implantation [4, 11–16, 21]. Ikiz Bozsoy and Yücel [5] and Sparreboom et al. [6] similarly reported superior performance among bilateral users. Li et al. [22] reported that after one year of bilateral use, children required approximately 2.7 dB lower signal-to-noise ratio (SNR) to achieve comparable Hearing-in-Noise Test scores.

Heterogeneity was moderate to high (I² = 50–60%) due to variability in test paradigms, SNRs and sample characteristics (age at second CI, bilateral use duration). Most speech-in-noise studies used fixed SNRs between + 5 and + 10 dB, although some employed adaptive procedures (Table 1).

A random-effects model was used to account for this variability. The pooled standardized mean difference (SMD) was + 0.70 in favour of BiCI (95% CI + 0.45 to + 0.94, *p* < 0.001). Subgroup analysis showed lower heterogeneity in noise-only test conditions (I² < 25%).

Funnel plot inspection and Egger´s test (*p* = 0.063) suggested no strong publication bias, although small-study effects could not be full excluded.

Shorter inter-implant delays (< 12 months) were associated with larger improvements in speech-in-noise compared with longer delays (> 36 months), and children implanted before 3 years of age showed greater bilateral benefit.

### Sound localisation outcomes

**Fig. 3:**
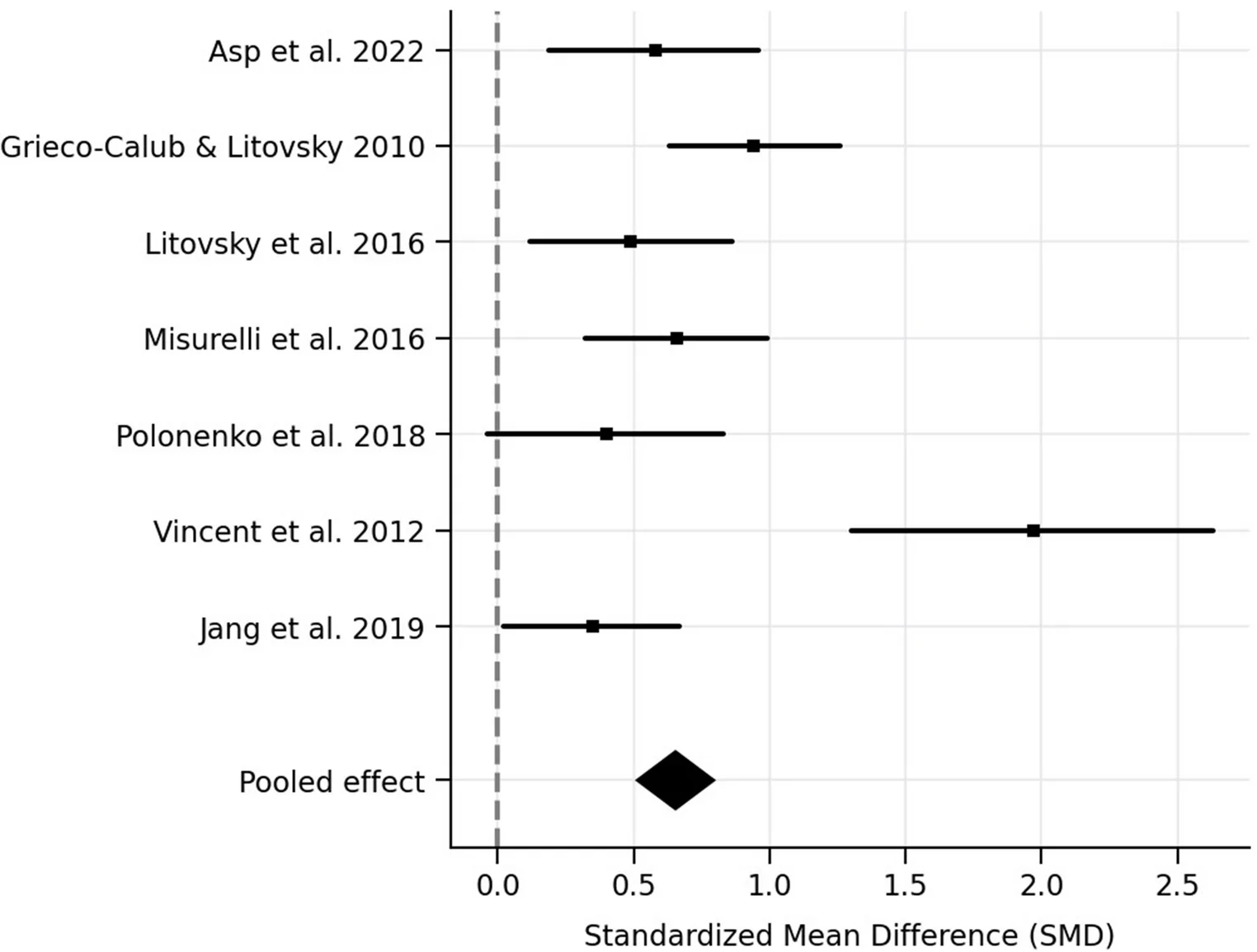
Forest plot of standardized mean differences (SMDs) comparing bilateral cochlear implantation (BiCI) with unilateral cochlear implantation (UniCI) for **sound localisation and spatial‑hearing outcomes**. Localization accuracy was assessed using root mean square (RMS) error, angular deviation, lateralisation accuracy, or equivalent metrics, transformed so that positive SMD values indicate better performance with bilateral implantation. The pooled effect (diamond) represents the fixed‑effect meta‑analytic estimate. Pooled sound localisation effect: SMD = 0.65 (95% CI = 0.51 to 0.80)

Seven studies [20, 21, 23–27] contributed localisation data (Fig. 3). Unilateral CI users demonstrated root-mean-square (RMS) localisation errors of 35–45°, whereas bilateral users averaged 20–30°, representing an improvement of approximately 10–15°. Meta-analysis showed a pooled SMD of + 0.74 (*p* < 0.001) with low heterogeneity (I² 0–10%). Simultaneous implantation or short inter-implant delay was associated with superior localisation performance compared with long-gap sequential implantation, although both groups showed significant benefit. Considerable methodological heterogeneity was observed in sound localization setups, with loudspeaker arrays ranging from 3 to 12 units and different metrics used (RMS error vs. percent correct), which may impact the comparability of these outcomes.

### Language and developmental outcomes

**Fig. 4:**
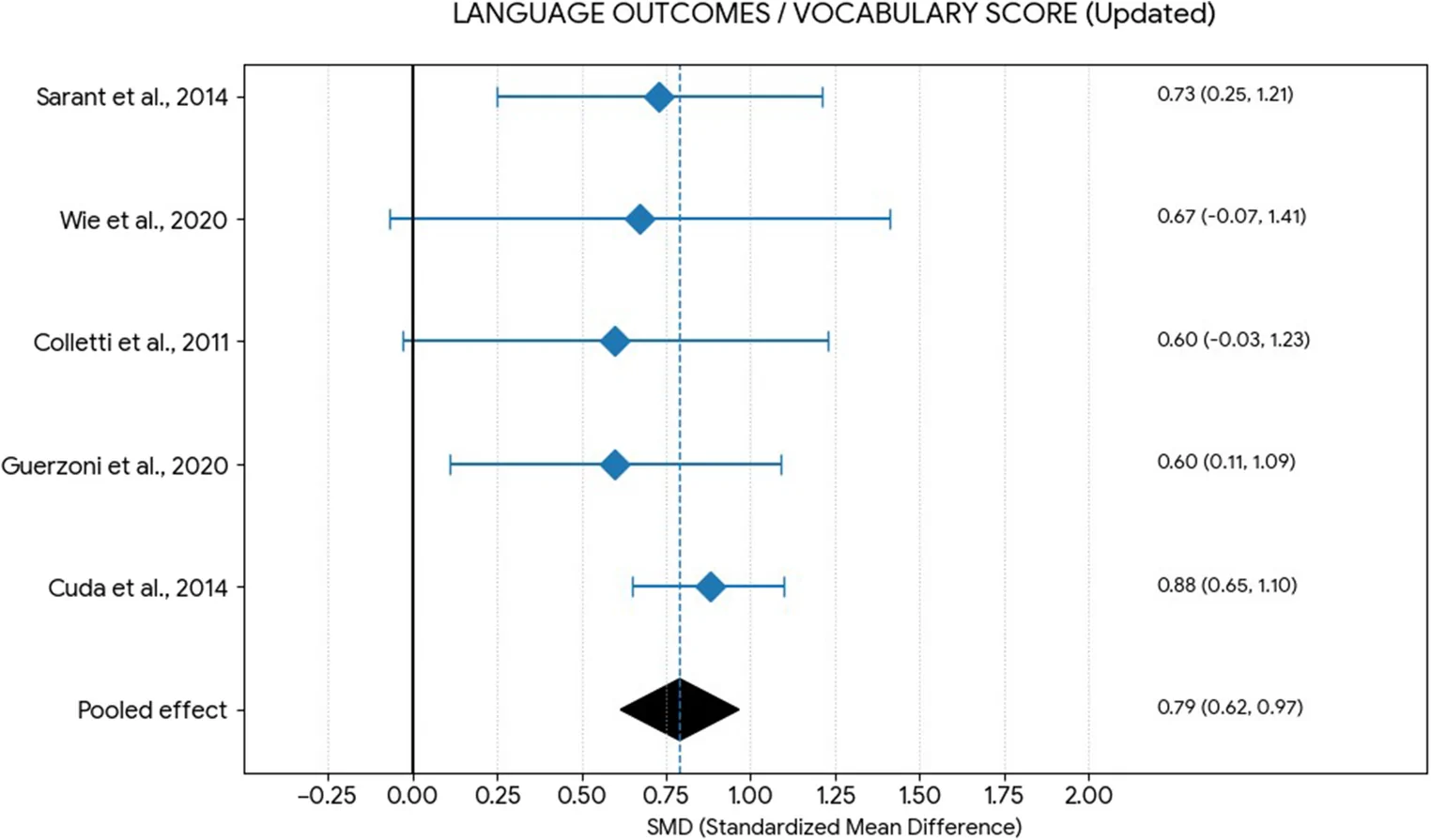
Forest plot of standardised mean differences (SMD) in PPVT vocabulary scores comparing bilateral versus unilateral cochlear implant use in children. Each study is represented with a point estimate (diamond) and 95% confidence interval (horizontal line). Diamonds are proportional to study weight

While auditory measures showed a clear bilateral advantage, language outcomes were more influenced by the timing of the first intervention. Twelve studies reported spoken language outcomes. Cuda et al. [17] demonstrated that children receiving their first CI before 12 months achieved significantly higher vocabulary and sentence complexity than those implanted later, highlighting the importance of the early auditory ‘critical period’. However, when specifically comparing bilateral to unilateral users, the meta-analysis of four studies [2, 26, 28, 29] showed a pooled SMD of + 0.65 (95% CI 0.38–0.93) in favor of BiCI (Fig. 4) indicating approximately half a standard deviation higher receptive vocabulary in bilateral users. Wie et al. [26], Sparreboom et al. [30], and Colletti et al. [28] all reported higher receptive vocabulary and comprehension scores in bilateral users. Other cohorts [2, 6] reported more advanced vocabulary and grammar in BiCI recipients by early school age. Early-implanted children can achieve age-appropriate spoken language by school age [31].

Twelve studies reported spoken language outcomes. Large cohort studies and meta-analyses showed a trend toward superior vocabulary, language comprehension, and reading outcomes in BiCI compared with UniCI users although findings were more heterogeneous than auditory outcomes [2], 21– [16, 26, 28–30].

Phonological awareness and emergent literacy also favoured BiCI [32–34]. Device data-logging studies showed that consistent bilateral use predicted superior linguistic outcomes [18, 29].

Executive functioning and working memory, which support language learning, are also better developed in children with effective auditory input [35].

### Secondary outcomes

Twelve studies reported surgical outcomes. Sbeih et al. [36], Saleeb et al. [37], and Asghari et al. [38] reported comparable complication rates per ear for BiCI and UniCI, with major complications occurring in approximately 2–3% and minor complications in 5–10%. Per-implant complication rates were similar between unilateral and bilateral surgery [39]. Simultaneous BiCI was well tolerated and did not increase perioperative morbidity [9, 36–39].

Vestibular Outcomes.

Four studies evaluated vestibular function. Shen et al. [7] reported otolith dysfunction in some children with inner-ear malformations. Vibert et al. [40] found dizziness in 15% of bilateral recipients without long-term gait impairment. These two studies used objective vestibular testing (calorics, vHIT, and/or VEMP) [7, 40], whereas other studies relied on parental or questionnaire-based assessments. Most children retained vestibular function in at least one ear. Parental reports of balance or dizziness were described by Galvin et al. [41] and Cejas et al. [42].

### Quality of life

Multiple studies using SSQ, LittlEars and CCIPP reported higher parental ratings of communication, spatial hearing, and daily functioning in BiCI compared with UniCI users [35, 43–45]. Everyday listening performance also improves significantly after receipt of the second implant [41]. Device rejection occurred in approximately 5–10% of cases and was usually transient [9, 44]. Cejas et al. [42] found no significant QoL difference between early and late BiCI. Cross-country variation in parental ratings was observed [43, 46].

An adapted Newcastle–Ottawa Scale was used to assess risk of bias across observational studies, focusing on cohort comparability, assessor blinding, and completeness of follow-up; individual study ratings are presented in Table 2.

**Table 2:** Risk of bias assessment conducted using the Newcastle-Ottawa Scale (NOS) for cohort studies.

Author (Year)	Study Design	Sample Size (*N*)	Outcomes Assessed	Measurement Tools	Effect Size (SMD, 95% CI)	NOS Score
Chen et al. (2024) [11]	Within-subject cohort	124	Speech in noise	Custom Mandarin sentence test	0.60 (0.41, 0.79)	**8**
Galvin & Mok (2016) [41]	Within-subject cohort	14	Speech in noise	Custom sentence-in-noise test	0.77 (0.17, 1.37)	**7**
Cuda et al. (2014) [18]	Cross-sectional cohort	30	Speech development	MCDI at 36 months	0.88 (0.65, 1.10)	**7**
Ikiz Bozsoy & Yücel (2024) [5]	Cross-sectional cohort	50	Speech in noise	HINT-C (Turkish)	1.10 (0.51, 1.69)	**8**
Jacobs et al. (2016) [12]	Cross-sectional cohort	49	Speech in noise	AAST	0.93 (0.32, 1.54)	**8**
Johnstone et al. (2010) [13]	Within-subject cohort	16	Speech in noise	Custom spatialized speech test	0.74 (0.19, 1.29)	**7**
Li et al. (2023) [22]	Within-subject cohort	20	Speech in noise	Mandarin HINT	0.66 (0.18, 1.14)	**7**
Puechmaille et al. (2020) [24]	Within-subject cohort	63	Speech in noise	Custom protocol (BiCI vs. HA)	0.25 (0.00, 0.50)	**8**
Sparreboom et al. (2011) [6]	Cross-sectional cohort	38	Speech in noise	Sentence in noise (% correct)	1.20 (0.40, 2.00)	**8**
Torkildsen et al. (2019) [25]	Cross-sectional cohort	26	Speech in noise	HINT (Norwegian)	0.63 (−0.16, 1.42)	**7**
Grieco-Calub & Litovsky (2010) [21]	Cross-sectional	38	Sound localization	Speaker-array RMS error	0.94 (0.63, 1.26)	**7**
Litovsky et al. 2016) [48]	Within-subject cohort	13	Sound localization	Speaker-array RMS	0.49 (0.12, 0.86)	**8**
Misurelli et al. (2016) [15]	Within-subject cohort	13	Sound localization	Custom 7-speaker array	0.66 (0.32, 0.99)	**8**
Polonenko et al. (2018) [50]	Within-subject cohort	9	Sound localization	Multi-speaker localization	0.40 (−0.04, 0.83)	**7**
Vincent et al. (2012) [16]	Cross-sectional	18	Lateralization	% correct discrimination	1.97 (1.30, 2.63)	**7**
Jang et al. (2019) [17]	Cross-sectional	45	Sound localization	Angular deviation	0.35 (0.02, 0.67)	**7**
Sarant et al. (2014) [2]	Cohort (between)	91	Receptive vocabulary	PPVT-4	0.73 (0.25, 1.21)	**8**
Wie et al. (2020) [26]	Cohort (between)	30	Receptive vocabulary	PPVT-4	0.67 (−0.07, 1.41)	**8**
**Colletti et al.** (2011) [29]	Cohort (between)	40	Receptive vocabulary	Italian PPVT-R	0.60 (−0.03, 1.23)	**8**
Guerzoni et al. (2020) [31]	Cohort (between)	89	Receptive vocabulary	Italian PPVT	0.60 (0.11, 1.09)	**8**

The scale evaluates three categories: Selection (representative nature of the BiCI cohort, selection of UniCI controls, ascertainment of exposure, and demonstration that the outcome was not present at start; max 4 stars); Comparability (control for the most critical confounders: age at implantation and inter-implant delay; max 2 stars); and Outcome (assessment of auditory/language results via objective standardized testing, sufficient follow-up length, and adequacy of follow-up/retention; max 3 stars). Quality Thresholds: Total scores range from 0 to 9. Following standard criteria, studies are categorized as: High Quality: 7–9 stars; Moderate Quality: 5–6 stars: Low Quality: 0–4 stars. Max = máximum

## Discussion

This systematic review and meta-analysis demonstrate that bilateral cochlear implantation provides meaningful advantages over unilateral implantation in key auditory and developmental domains. Improvements were most evident for speech perception in noise and sound localisation, domains that depend on binaural processing and are critical for real-world listening.

Binaural hearing allows access to interaural timing and level cues [47, 48], supporting spatial hearing, speech segregation, and listening efficiency. These mechanisms likely explain why bilateral users consistently outperform unilateral peers in noisy environments and spatial tasks. Earlier bilateral stimulation is associated with more symmetric cortical development and more effective binaural integration [49], although benefits were also observed in children implanted after longer delays.

Language outcomes showed a consistent but more heterogeneous advantage for BiCI, reflecting the multifactorial nature of language development. Improved access to speech in complex environments likely enhances incidental learning and classroom participation, indirectly supporting vocabulary, literacy, and academic readiness.

From a safety perspective, bilateral implantation did not increase surgical risk compared with unilateral implantation. Vestibular effects were generally mild and transient, and quality-of-life measures indicated broad functional benefits across communication, independence, and social engagement.

## Limitations

The main limitation is the observational design of most included studies, with potential confounding. Outcome measures and reporting were heterogeneous, limiting comparability. Importantly, inter-implant delay was inconsistently reported and usually only at the study level, precluding calculation of pooled patient-level delay distributions.

Additional sources of heterogeneity included variability in speech-in-noise paradigms and signal-to-noise ratios, use of free-field versus ear-specific testing, inconsistent reporting of contralateral hearing-aid use in unilateral CI comparisons, and wide variation in vestibular assessment methods ranging from objective testing to parental report. These factors limit direct comparability across studies and should be considered when interpreting pooled effect sizes.

Regarding the timing of the second implant in sequential cases, our analysis reveals a clear distinction in outcomes based on the inter-implant interval. The majority of the studies showing optimal bilateral benefits reported a relatively short delay of 1–3 years [8, 23, 26, 29]. In contrast, consistent with the concept of a critical period for bilateral auditory development, cohorts that included long-gap sequential cases exceeding 5 years [18, 19, 22, 44] often exhibited more heterogeneous outcomes, particularly in complex sound localization tasks.

Language outcomes are also influenced by family environment and socioeconomic factors, which could not be controlled for in this meta-analysis [50].

Despite the observational nature of most included studies, consistent cohort characterization and generally complete follow-up contributed to moderate to high methodological quality overall (Table 2); however, causal inferences remain limited.

### Clinical implications

The evidence supports bilateral implantation—preferably simultaneous or with short inter-implant delay—as standard care for children with bilateral profound hearing loss, provided appropriate audiological and speech-language rehabilitation is delivered.

### Future directions

Future research should adopt harmonised outcome measures and follow children into adolescence to capture educational, psychosocial, and cognitive outcomes. Advances in binaural CI processing may further enhance spatial hearing and speech-in-noise performance.

## Supplementary Information

Below is the link to the electronic supplementary material.

Supplementary Material 1
