# Supplementary material for: Benefits of bilateral cochlear implantation in children: a systematic review and meta-analysis
Source: Eur Arch Otorhinolaryngol. 2026 May 21;283(9):5547–56. doi: 10.1007/s00405-026-10302-z (PMC13615015; doi:10.1007/s00405-026-10302-z)
Supplement: Supplementary file 1 [file 405_2026_10302_MOESM1_ESM.docx]

Supplementary table 1. Qualitative analysis

| **Study (Ref #, Year)** | **Study design** | **Sample Size (N)** | **Age (mean or range)** | **Outcomes Assessed** | **Measurement Tools** |
| --- | --- | --- | --- | --- | --- |
| **[1] Yoshimura et al., 2024** | Narrative review | 153913 | newborn | Epidemiology, aetiology, diagnosis of congenital HL | Prevalence, severity, laterality, and aetiology of congenital hearing loss |
| **[3] Litovsky & Gordon, 2016** | Narrative review | N.A. | Pediatric populations (typically ≥ 2 years; normal‑hearing spatial hearing mature ~4–5 years) | Spatial hearing, sound localization, spatial release from masking, binaural sensitivity (ITD/ILD), effects of auditory deprivation and plasticity | Behavioral localization tasks (MAA, RMS error); speech‑in‑noise paradigms (SRM); direct electrical stimulation using research processors; electrophysiological measures (e.g. binaural interaction component) |
| **[4] Cullington et al., 2017** | National cohort | 1001 children (465 simultaneous BiCI; 536 sequential BiCI) | Simultaneous BiCI: median 2.1 years (7% < 1 year); Sequential BiCI: inter‑implant interval 0.1–14.5 years | Sound localization; speech recognition in noise; spatial release from masking | Free‑field sound localization tasks; speech recognition in noise testing |
| **[6] Sparreboom et al., 2015** | Longitudinal observational cohort with matched control group | 29 sequential BiCI children; 9 UniCI controls | Age at second CI: 2.8–8.5 years | Speech perception in quiet and noise; lateralization; minimum audible angle | Speech reception tests (quiet and noise); lateralization task; minimum audible angle (speaker array at ±42°) |
| **[7] Shen et al., 2025** | Observational clinical study | 78 children with  SNHL (39 with inner  ear malformations;  39 controls) | Mean age 5.7 ± 4.1 years | Otolith function; balance; gross motor development before and after CI | Cervical and ocular vestibular‑evoked myogenic potentials (cVEMP, oVEMP); gross motor developmental milestones |
| **[8] Sarant et al., 2019** | International prospective longitudinal cohort | 59 older adults (baseline); 20 with 18‑month follow‑up | 61–89 years (mean 72.3 ± 6.8 years) | Cognitive function; speech perception; quality of life; psychosocial and lifestyle factors | Cognitive: Cogstate Brief Battery, Groton Maze Learning Test (GMLT), MMSE (screening only); Audiology: pure‑tone audiometry, CVC words, speech reception thresholds in noise; Questionnaires: APHAB, HUI‑3, HADS, IPAQ, LSNS, loneliness scales |
| **[9] Thomas et al., 2017** | Retrospective clinical cohort | 21 children with congenital single‑sided deafness | Age at implantation: 10 months–11.3 years | Speech recognition in noise; binaural hearing effects (head shadow, summation, squelch); lateralization; subjective hearing benefit | German Oldenburg Sentence Test for Children (OlKiSa); lateralization tasks; Speech, Spatial and Qualities of  Hearing Scale (SSQ) questionnaires |
| **[14] Forli et al., 2023** | Retrospective observational cohort (sequential BiCI) | 22 children | CI1: 10–48 mo; CI2: 28–140  months | Speech perception (quiet, noise); SRT;  binaural SRT gain; implant usage | Disyllabic word recognition; OLSA matrix  sentence test (SRT); device datalogging |
| **[19] Ching et al., 2014** | Population‑based prospective longitudinal cohort | 165 children with CIs (14 UniCI; 56 CI + HA;  95 BiCI) | Assessed at **5 years of age**  (subset reassessed at 9 years) | Language ability; receptive vocabulary; phonological awareness; early reading skills; nonverbal cognition | PLS‑4 (receptive & expressive language); PPVT‑4  (receptive vocabulary); CTOPP (elision, sound matching, blending); Woodcock‑Johnson III® Diagnostic Reading  Battery (word identification, word attack, letter  knowledge); Wechsler Nonverbal Scale of Ability |
| **[20] Sharma et al., 2017** | Retrospective observational cohort | 180 children with unilateral CI | Age at implantation ≤ 4 years (mean 2.7 years; range 0.7–4 years) | Auditory perception; auditory integration; speech intelligibility; effect of age at implantation  and socioeconomic factors | Categories of Auditory Performance (CAP); Meaningful Auditory Integration Scale (MAIS); Speech Intelligibility Rating (SIR) |
| **[23] Killan et al., 2019** | Retrospective observational cohort | 127 children with bilateral cochlear implants (65 simultaneous;  62 sequential) | ≥ 4 years at testing; ≥ 12 months post‑second implant; sequential group median inter‑implant interval = 58 months  (range 3–143) | Sound localization accuracy; effects of inter‑implant interval, onset of deafness, CI manufacturer, and time since second  implant | Free‑field sound‑source localization task (5‑loudspeaker  array at −60° to +60° azimuth); root mean square (RMS)  localization error |
| **[27] Jaisinghani et al., 2024** | Retrospective observational cohort (sequential BiCI) | 60 children with sequential bilateral cochlear  implants (18 with longitudinal  follow‑up) | Age at 2nd CI: 1.1–19.0 years (four age groups: 1.1–5.0; 5.1–10.0;  10.1–14.0; 14.1–19.0) | Sound localization accuracy; effects of age  at 2nd CI, inter‑implant interval, prior HA  experience, and BICI experience | Free‑field sound localization task using speech‑shaped  noise; mean absolute error (MAE, degrees) |
| **[28] Sparreboom et al., 2011** | Long‑term longitudinal cohort with matched unilateral CI reference group | 24 children with  sequential bilateral  CIs; 26 children with  unilateral CI | Mean age at testing: 10.8 years (BiCI) vs 9.8 years (UCI); mean age at  CI2: 5.1 years | Spatial hearing; speech recognition in quiet  and noise; receptive vocabulary;  educational outcomes | Minimum Audible Angle (MAA); Dutch NVA children’s  speech test; PPVT‑III‑NL (Dutch version) |
| **[30] Dettman et al., 2016** | Prospective  longitudinal  multicentre cohort | 403 children implanted < 6 years  (151 implanted  < 12 months) | Age at CI: < 12 mo to 72 mo; followed to school entry and late primary/secondary | Speech perception; receptive &  expressive language; speech production;  developmental trajectories | CNC words & phonemes; BKB sentences; PLS‑4/5;  PPVT‑III/IV; CELF; DEAP |
| **[32] Bruijnzeel et al., 2017** | Retrospective  observational cohort | 54 children implanted < 36 months  (unilateral and bilateral CIs) | Median age at CI: 22.9 months (range 8.5–34.1); followed  5 years post‑CI | Long‑term speech perception; probability of ceiling performance; effect of age at implantation | Open‑set CVC(A) word lists in quiet; raw scores and ceiling analysis (> 95%) |
| **[34] Nittrouer et al., 2012** | Cross‑sectional  observational cohort | 27 children with CIs;  17 NH controls;  8 HA users | Kindergarten  (post‑kindergarten entry) | Emergent literacy (word reading, comprehension, fluency); phonological awareness; oral  language; executive functioning; effects of  age at implantation and bimodal experience | Qualitative Reading Inventory‑4; phonological awareness tasks; PLS‑4; expressive vocabulary tests; narrative analysis; verbal short‑term memory; rapid serial naming |
| **[35] Kronenberger et al., 2014** | Cross‑sectional  observational cohort | 64 long‑term CI users; 74 normal‑hearing  controls | 7–27 years; mean  CI use ≈ 12 years | Executive functioning (EF); speech  perception; receptive and expressive  language;  EF–language associations | EF battery (WISC/WJ tasks, TOVA); PPVT‑4; CELF‑4;  speech perception tests (LNT, AVLNST, HINT‑C) |
| **[37] Saleeb et al., 2025** | Retrospective  observational cohort | 1024 CI recipients; 45 syndromic cases | Mostly prelingual children  < 5 years | Syndromic prevalence; inner/middle ear malformations; intra‑operative difficulties; descriptive audiological outcomes | Surgical challenges and feasibility of CI in syndromic  hearing loss |
| **[38] Asghari et al., 2023** | Retrospective  multicentre cohort | 448 children (86 < 12 mo; 362 12–24 mo) | Mean: 9.7 mo vs 18.2 mo | Surgical complications; auditory  performance; speech intelligibility | CAP; SIR |
| **[39] Cullen et al., 2008** | Retrospective  multicentre cohort | 952 pediatric CI  surgeries; 93 children requiring revision | Children with long‑term CI use | Indications for revision; device failure;  surgical complications; post‑revision  performance | Long‑term device reliability and revision surgery |
| **[40] Vibert et al., 2001** | Prospective  observational study | 15 CI recipients | Mixed pediatric & adult | Canal and otolithic vestibular function; postoperative vertigo | Vestibular impact and preservation after CI |
| **[42] Cejas et al., 2023** | Prospective  longitudinal  multicentre cohort | 188 children with CIs | Implanted <5 y; assessed  at 9–13 y post‑CI | Academic achievement; spoken language;  quality of life | Long‑term educational and QoL outcomes; effect of early implantation |
| **[44] Gundacker et al., 2023** | Pediatric cohort | 92 children | 2-19 years | Generic QoL (AQoL‑6D) ; Hearing‑related QoL (SSQ12); Audiology (PTA, HSM sentence test); Early vs late implantation comparisons | AQoL‑6D; SSQ12; Pure tone audiometry (PTA); HSM sentence test |
| **[45] Sparreboom et al., 2012** | Prospective  longitudinal cohort with matched  unilateral CI reference  group | 30 children with sequential bilateral CIs;  9 children with  unilateral CI | Median age at CI1: 2.0 years (range 0.9–2.7); median  age at CI2: 5.0 years  (range 2.4–8.5) | Health‑related quality of life (generic and disease‑specific); longitudinal bilateral  benefit over time | Generic QoL: VAS, HUI3, PedsQL; Disease‑specific  QoL: GCBI, SSQ, NCIQ |
| **[46] Kumar et al., 2015** | Cross‑sectional observational study | 33 families (for 32 children) | Mean age at CI activation: 2.47 ± 1.85 years  Mean CI experience: 7.47 ± 2.80 years; | Condition‑specific health‑related quality of life (HRQoL) | CI‑specific parent‑proxy HRQoL instrument (8 psychosocial domains, 5‑point Likert scale) |
